# Edible Coating Based on Alginate Nanoparticles Containing Menthol and *Zataria multiflora* Essential Oil: Effect on the Shelf Life of Chicken Thigh in the Refrigerator

**DOI:** 10.1002/fsn3.4684

**Published:** 2024-12-19

**Authors:** Ali Ranjbar, Mahmoud Osanloo, Mojdeh Safari, Zahra Eskandari, Ehsan Safari, Amene Nematollahi

**Affiliations:** ^1^ Department of Medicine, School of Medicine Fasa University of Medical Sciences Fasa Iran; ^2^ Student Research Committee Fasa University of Medical Sciences Fasa Iran; ^3^ Department of Medical Nanotechnology, School of Advanced Technologies in Medicine Fasa University of Medical Sciences Fasa Iran; ^4^ Noncommunicable Diseases Research Center Fasa University of Medical Sciences Fasa Iran; ^5^ Finetech in Medicine Research Center, School of Medicine Iran University of Medical Sciences Tehran Iran; ^6^ Department of Food Safety and Hygiene, School of Health Fasa University of Medical Sciences Fasa Iran

**Keywords:** active packaging, alginate, chicken thigh, edible coating, essential oil, nanoparticles

## Abstract

Chicken thigh is a popular and widely consumed meat product. However, its high moisture content and susceptibility to microbial spoilage limit its shelf life. To address this issue, we investigated the efficacy of an edible coating based on alginate nanoparticles (AlgNPs) containing menthol, *Zataria multiflora* essential oil (EO), or their combination for extending the shelf life of chicken thigh. The nanoparticles were prepared through ionic gelation methods; their particle size was obtained as 87–205 nm with PDI values of < 0.3 and SPAN values of < 1. The AlgNPs containing both menthol and the EO showed the most potent antimicrobial activity (the lowest counts of pseudomonas, aerobic mesophilic, and yeast‐mold), during cold storage. This coating treatment showed also the highest antioxidant activities, with the lowest thiobarbituric acid reactive substances (TBARS), pH and total volatile basic nitrogen (TVBN) values. Moreover, this coating significantly (*p* < 0.05) showed the highest sensory quality of chicken thighs, as evidenced by the higher sensory scores for texture, odor, color, and overall acceptability. Therefore, the edible coating based on AlgNPs containing menthol and *Z. multiflora* EO is an effective and promising approach for extending the shelf life of chicken thighs.

## Graphical abstract

Alginate nanoparticles containing menthol and *Zataria multiflora* EO appear as an edible new active packaging method for guaranteeing the realistic preservation of fresh thigh chicken meat in cold storage in light of these convincing results.

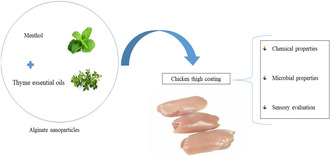

## 1 Introduction

Chicken meat is a wonderful source of animal protein of low lipid content and excessive organic value, including all the critical amino acids and unsaturated fatty acids required for a human regimen (Salama, Belih, and Khedr 2023). As one of the most famous meals worldwide, chicken meat consumption has improved dramatically in many nations in recent years, accounting for almost 30% of global meat consumption (Cao et al. 2022). Customers often choose fresh chicken meat compared to frozen meat (Hematizad et al. 2021). However, fresh chicken meat is at risk of microbial and chemical spoilage because of its excessive range of moisture and nutrients at the appropriate conditions for these reactions. Therefore, the demand for methods that increase the shelf life of fresh chicken meat is growing. Freezing, using chemical preservatives, and packaging or their combinations are the most common methods for spoilage retarding of chicken meat. Each of the mentioned methods has disadvantages (Xu et al. 2022).

When it comes to chemical preservatives, they may be unsafe because they usually have potential carcinogenic, toxicogenic, and teratogenic outcomes (Yu, Chin, and Paik 2021). Considering packaging, it is worth mentioning that chemicals derived from synthetic substances may be a remarkable danger to human life (Abbasi et al. 2021). Furthermore, these synthetic plastics, derived from petroleum, do not decompose in the environment and can endanger the ecosystem (Abbasi et al. 2021). To resolve the noted environmental problems of plastic substances, edible films and coatings (based on lipids, proteins, polysaccharides, or their combination) have been increasingly used in food packaging. The increasing interest in using this type of packaging could be due to their degradability properties, absence of synthetic pollutants, safety for human health, and the possibility of incorporating natural preservatives, such as herbal essential oils (EOs), during production. Edible coatings and films can also increase the quality of foods by preventing physical damage, in addition to controlling the transport of antimicrobial agents and transferring moisture and oxygen (Nikravan et al. 2024; Tavassoli‐Kafrani, Shekarchizadeh, and Masoudpour‐Behabadi 2016).

EOs are natural substances extracted from numerous plants such as thyme, mint, oregano, etc. These compounds can inhibit or limit the growth of harmful bacteria with their specific functional components. In this regard, the antibacterial properties of EOs are usually attributed to oxygenated terpenoids (such as alcohol and phenolic terpenes; Osanloo, Ghaznavi, and Abdollahi 2020; Osanloo et al. 2022). It is noteworthy that EOs are generally recognized as safe (GRAS) under the Food and Drug Administration of United States (USFDA) regulations and are also classified as being safe as food additives at concentrations below 2 mg/kg body weight per day, regarding the European Union (EU) recommendation. Therefore, EOs are added to food products as flavoring, antioxidant, and antimicrobial agents under “natural green” preservatives (Roozitalab et al. 2022; Teshome et al. 2022). However, EOs are highly volatile and have low water solubility properties. Moreover, they have a sweet smell, a strong aroma, and an oily nature, which sometimes causes problems in their use. Hence, EOs can be incorporated into edible coatings and films to achieve active packaging with more benefits to solve the noted problems. One of the important advantages of this approach is that the EOs cannot easily be removed from the edible packaging, and so remain in high concentrations around the product for a long time during storage (Esmaeili et al. 2022; Osanloo, Jamali, and Nematollahi 2021). Moreover, in this case, a smaller amount of these compounds is needed than adding them directly to the foods, which will prevent the creation of a strong aroma in the product (may be unpleasant for many consumers; Shahidi and Hossain 2022; Valizadeh et al. 2023).

*Zataria multiflora* Boiss, a member of the Labiatae or Lamiaceae family, known as thyme‐e‐Shirazy (in Persian), grows wild in Asia, including the central and southern regions of Iran, Pakistan, and Afghanistan (Bahrami et al. 2023; Pateiro et al. 2021). It is widely used in foods (especially meat and dairy products) as a flavoring and antimicrobial agent. *Z. multiflora* EOs have successfully demonstrated antibacterial and antifungal activities due to the presence of high levels of polyphenols (like thymol and carvacrol), showing antioxidant and antimicrobial activities even at low concentrations (Mehdizadeh and Langroodi 2019). Menthol, a cyclic terpene alcohol, is widely used as an allowed and pleasant additive in foods, cosmetics, and medicine. In addition to being cheap and non‐toxic, menthol has antifungal, antioxidant, and antimicrobial activities, according to various studies (Hazer and Ashby 2023; Mehdizadeh and Langroodi 2019).

Edible films and coatings containing nanoparticles of EOs can alleviate some of the concerns of the food packaging sector due to their biological and size‐dependent properties (such as higher surface area compared to larger particles; Jafarzadeh et al. 2023). These very small materials can increase the shelf life of food and enhance food safety and security by reducing food waste due to chemical and microbial spoilage. Despite the strict prohibition on using metal nanoparticles in food packaging (by the EU), the nanoparticles could be made from non‐metal edible components such as proteins, polysaccharides, and EOs (Osanloo et al. 2023). Alginate is a polysaccharide that was first discovered in seaweed and is widely used as a coating on the surface of food to prevent the growth of microorganisms (Severino et al. 2019). It is also a biopolymer commonly used to encapsulate various compounds, including EOs, due to its availability, low cost, ease of gelation, and non‐toxicity nature (Baek, Lee, and Oh 2021; Rahnemoon et al. 2021).

The antimicrobial and antioxidant properties of EOs incorporated into the edible films and coatings have been proven by several studies like, chitosan coatings incorporated with propolis extract and *Z. multiflora* on chicken breast meat, chitosan coating containing *Z. multiflora* EO on chicken fillets (Bahmani and Abolfathi 2022), sodium alginate in combination with *Z. multiflora* on fresh pistachio ( *Pistacia vera* L.; Hashemi, Shakerardekani, et al. 2021), sodium alginate and chitosan coating combined with three different EOs on rainbow trout fillets (Raeisi et al. 2020). However, as far as we researched, there is not yet a study on using menthol and *Z. multiflora* EO in combination with sodium alginate nanoparticles for manufacturing a new active packaging for chicken thigh preservation. Therefore, given the importance of chicken in the diet, the cheapness of alginate, and the possible synergistic effectiveness of EOs in nano size, the main aim of the present study is to investigate the microbial, chemical, and sensory characteristics of chicken thigh‐coated with sodium alginate nanoparticles containing menthol and *Z. multiflora* EO during 16 days of storage at 4°C.

## 2 Materials and Methods

### 2.1 Materials

Freshly purchased chicken thighs from a local market in Fasa, Fars province, Iran, were immediately transported to the laboratory on ice. After skin removing and thorough washing, the chicken thighs were cut, with a sterile knife, into pieces with thichness of about 4 cm and weight of approximately 50 g. All culture media, including plate count agar (PCA), Dichloran‐rose bengal chloramphenicol agar (DRBC), cetrimide fuzidine cephaloridine agar (CFC), as well as peptone water (PW), Menthol, Tween 20, Tween 80, 1‐butanol, Trichloroacetic acid (TCA), Magnesium oxide (MgO), Boric acid, and Hydrochloric acid were obtained from Merck Company (Darmstadt, Germany). Calcium chloride dihydrate (CaCl2) and sodium alginate were also obtained from Sigma‐Aldrich Corporation (Steinheim, Germany). EOs of *Z. multiflora* were purchased from Zardband Pharmaceuticals Company (Iran). It should be mentioned that distilled water was used in all experiments.

### 2.2 GC–MS Analysis

The gas chromatography device utilized was an Agilent 6890 type, featuring a 30 m long column with an inner diameter of 0.25 mm and a layer thickness of 0.25 μm of the BPX5 type. To identify the components of the EO, 1 μL of sample, diluted in n‐hexane, was injected into the GC/MS machine. The column's temperature program was set as follows: the oven's initial temperature was 50°C, held for 5 min, followed by a thermal gradient of 3°C per min, raising the temperature to 240°C. Then, the temperature was increased at a rate of 15°C per min up to 300°C, with a final hold at this temperature for 3 min. The overall study time was 75 min. The injection chamber temperature was set at 250°C with a split ratio of 1–35, and helium gas was used as the carrier gas at a flow rate of 0.5 mL/min. The mass spectrometer employed was an Agilent 5973 model with an ionization voltage of 70 eV, using the EI ionization method, and the ion source temperature was 220°C. The scan range was set from 40 to 500, and ChemStation software was used for analysis. The spectra were identified by their retention index and compared with those in reference books and articles, alongside the mass spectra of standard compounds and the information in the computer's library (Adams 2001; McLafferty et al. 1989).

### 2.3 Preparation of Nanoparticles' Coatings

Four coating solutions were prepared by the hotplate magnetic stirrer with a speed of 2000 rpm (alpha, Iran) as follows: (1) Alginate nanoparticles alone without any additives (AlgNPs): 25 μL of tween 20 and 25 μL of tween 80 are added to 2.5 mL of alginate solution (0.5% w/v) and then placed in an ultrasonic device for 1 h. Then, 2.5 mL of CaCl_2_ solution (0.1% w/v) was added and stirred for 40 min to form nanoparticles. (2) AlgNPs containing menthol (AlgNPs–menthol): 12.5 mg of menthol crystal, 25 μL of tween 20, and 25 μL of tween 80 were added to 2.5 mL of alginate solution (0.5% w/v), and then placed in an ultrasonic device for 1 h. Then, 2.5 mL of CaCl2 solution (0.1% w/v) was added and stirred for 40 min to form nanoparticles. (3) AlgNPs containing *Z. multiflora* EO (AlgNPs–EO): 7 μL of tween 20 and 7 μL of tween 80 were added to 2.5 mL of alginate solution (0.5% w/v) and then placed in an ultrasonic device for 1 h. Then, 12.5 μL of *Z. multiflora* EO was added and stirred for 5 min. Finally, 2.5 mL of CaCl2 solution (0.06% w/v) was added and stirred for 40 min to form nanoparticles. (4) AlgNPs containing both of menthol and *Z. multiflora* EO (AlgNPs–menthol–EO): 12.5 mg of menthol crystal, 25 μL of tween 20, and 25 μL of tween 80 were added to 2.5 mL of alginate solution (0.5% w/v), and then placed in an ultrasonic device for 1 h. Then, 12.5 μL of *Z. multiflora* EO was added and stirred for 5 min. Finally, 2.5 mL of CaCl2 solution (0.1% w/v) was added and stirred for 40 min to form nanoparticles.

### 2.4 Characterization of Nanoparticles

The physicochemical properties of the prepared nanoparticles were characterized using dynamic light scattering (DLS) and attenuated total reflection fourier‐transform infrared spectroscopy (ATR‐FTIR). DLS (SZ‐100 series, HORIBA Scientific, Japan) was employed to determine the prepared nanoparticles' particle size, polydispersity index (PDI), particle size distribution (SPAN), and zeta potential. ATR‐FTIR spectroscopy (Bruker, Tensor II, Germany) was carried out to confirm successful EO and menthol loading in AlgNPs. Spectra of *Z. multiflora* EO, menthol, alginate powder, AlgNPs, AlgNPs–EO, AlgNPs–menthol, and AlgNPs–menthol–EO were recorded in the wavenumber range of 500–3500 cm^−1^ at room temperature.

### 2.5 Chicken Thigh Coating

Chicken thigh samples were divided into five groups (Mojaddar Langroodi, Nematollahi, and Sayadi 2021; Osanloo et al. 2023), including the uncoated control group and four chicken thigh samples coated with free alginate nanoparticles (AlgNPs), alginate nanoparticles containing menthol (AlgNPs–menthol), alginate nanoparticles containing *Z. multiflora* EO (AlgNPs–EO), and alginate nanoparticles containing both menthol and *Z. multiflora* EO (AlgNPs–menthol–EO). The treatment samples were immersed in their respective liquid coating solutions for 5 min. After drying at room temperature, the coated chicken thigh was carefully placed in a sterile polyethylene container and stored at 4°C for 16 days. Overall, using the full factorial design, regarding 5 treatment groups in 5 time intervals and 3 repetitions, 75 samples were prepared. Microbiological, chemical, and sensory evaluations were conducted at four‐day intervals (on days 0, 4, 8, 12, and 16) to assess the quality of the coated chicken thigh samples during cold storage.

### 2.6 Chemical Analysis

#### 2.6.1 pH

According to Baek et al. study, a 10 g sample of minced chicken thigh was homogenized with 90 mL of distilled water for 30 s using a high‐speed blender (in the ratio of 1/10). The pH of the resulting homogenate was measured at room temperature using a pH meter (GENWAY, 3510; Liu et al. 2020).

#### 2.6.2 Thiobarbituric Acid Reactive Substances (TBARS)

The thiobarbituric acid reactive substances (TBARS) assay was performed according to Sayyari et al. (2021) with minor modifications to quantify malondialdehyde (MDA), a marker of lipid oxidation. Briefly, 200 mg of minced chicken thigh sample was homogenized with a small volume of 1‐butanol, and the resulting mixture was adjusted to a volume of 25 mL using the same solvent. Subsequently, 10 mL of 0.2% (w/v) trichloroacetic acid (TCA) solution was added to 5 mL of the homogenized sample, and the mixture was incubated in a water bath at 95°C for 2 h. After cooling to room temperature, the absorbance of the solution was measured at 532 nm using a spectrophotometer. The TBARS value, expressed as mg MDA/kg chicken thigh, was calculated using Equation (1):

(1) $$\text{TBARS}=\left(50\times \left(A-B\right)\right)/m$$

*A*, *B*, and *m* represent the absorbance of the sample solution, the absorbance of the control solution, and the weight of the minced chicken thigh sample in milligrams, respectively (Sayyari et al. 2021).

#### 2.6.3 Total Volatile Basic Nitrogen (TVBN)

To calculate the TVBN amount, 10 g of minced chicken thigh was mixed with 2 g of magnesium oxide (MgO), 250 mL of distilled water, and a silicone oil droplet to prevent foaming. The mixture was transferred to a distillation apparatus containing 20 mL of 3% (w/v) boric acid solution. Methyl red and methylene blue indicators were added to the boric acid solution. After that, the solution was titrated with hydrochloric acid (HCl 0.01 N) until the endpoint was reached, as showed by the color change of the indicators. Finally, the TVBN amount was calculated by the volume of consumed HCl and was stated as mg of N/100 g chicken thigh according to Equation (2): (Goulas and Kontominas 2005).

(2) $$\text{TVBN}=\frac{\left(V1-V2\right)\times N\times 100\times 14\times 50}{W\times 5}$$

In this Equation, *V*1, *V*2, *N*, and *W* show volume of hydrochloric acid used for sample (mL), volume of hydrochloric acid used for blank (mL), normality of hydrochloric acid, and sample weight (g), respectively.

### 2.7 Microbiological Analysis

The microbial population of refrigerated chicken thighs was evaluated by Mojaddar Langroodi, Nematollahi, and Sayadi (2021) method, with minor alteration. One gram of minced chicken thigh was aseptically homogenized in 9 mL of 0.1% peptone water using a stomacher for 2 min. Decimal serial dilutions of the homogenate were prepared in 1% peptone water for all microbiological assays (the counts of Pseudomonas, aerobic mesophilic, and yeast mold).

#### 2.7.1 Total Aerobic Mesophilic

The total aerobic mesophilic counting was carried out by inoculating 0.1 mL of the homogenate solution, at specific dilutions, into duplicate sterile PCA by the surface spread plate method. Afterwards, the enumerartion were done after incubation of the plates for 24 h at 32°C (Mojaddar Langroodi, Nematollahi, and Sayadi 2021).

#### 2.7.2 Pseudomonas

For enumeration of Pseudomonas species, diluted samples were plated on CFC agar and incubated at 20°C for 24 h (Mojaddar Langroodi, Nematollahi, and Sayadi 2021).

#### 2.7.3 Yeast and Mold

The abundance of yeast and mold populations was determined using a surface spread plating method employing DRBC agar, a selective medium for these microorganisms. Subsequently, the inoculated plates were incubated at 25°C for a period of 72–120 h under dark conditions to facilitate optimal growth and enumeration of yeast and mold colonies (Mojaddar Langroodi, Nematollahi, and Sayadi 2021).

### 2.8 Sensory Evaluation

A panel of seven trained assessors conducted a sensory evaluation of chicken thigh samples. To ensure impartiality, the assessors were blinded to the experimental design, and the chicken thigh samples were identified using arbitrary numerical codes. The panelists assessed the samples' texture, odor, color, and overall acceptability using a 9‐point hedonic scale, where 9 represented favorable and 1 represented unfavorable. The assessors' satisfaction with their involvement in the study was also considered (Liu et al. 2020).

### 2.9 Statistical Analysis

All experiments in this study were conducted in triplicate, and the results are presented as mean values ± standard deviation (SD). The data were statistically analyzed using a two‐sample mean comparison test in STATA software (version 11). A *p*‐value < 0.05 was considered statistically significant.

## 3 Result and Discussion

### 3.1 Chemical Composition of *Z. multiflora*

The chemical constituents of *Z. multiflora* EOs were determined using GC–MS, revealing the presence of 26 compounds shown in Table 1. Thymol, carvacrol, and ortho‐cymen emerged as the main components, accounting for 26.48%, 38.71%, and 11.6% of the EO composition, respectively. Similar to our results, Osanloo et al. (2023) reported that thymol (25.2%) and carvacrol (30.2%) are the main components of *Z. multiflora* EOs (Osanloo et al. 2023). In another study conducted by Abbasi et al. (2021), carvacrol (36.62%) and thymol (17.86%) were determind as the main constituents of thyme EOs which is also agreed with the present results. However, the results of Bahrami et al. (2023) showed lower amounts of the identified components where the amount of o‐cymene, carvacrol and thymol in their study was 15.7%, 8.98% and 7.32%, respectively. Hematizad et al. (2021) also reported different concentration of carvacrol, linalool:, cymene: and thymol which were 48.19, 23.19, 5.52, and 4.13, respectively. These differences can be depended on diverse geographical condition, climatic alterations, plant stage, picking season, and EOs' extraction procedures (Abbasi et al. 2021).

**TABLE 1:** GC/MS analysis of *Zataria multiflora* EO.

No.	RT	%	Components	KI	Type
1	11.21	0.24	alpha‐Thujene	930	MH
2	11.6	3.17	alpha‐Pinene	939	MH
3	12.49	0.14	Camphene	954	MH
4	13.94	0.28	beta‐Pinene	979	MH
5	14.51	1.15	beta‐Myrcene	991	MH
6	15.99	0.83	alpha‐Terpinene	1017	MH
7	16.51	11.6	ortho‐Cymen	1029	MH
8	16.65	0.59	Limonene	1029	MH
9	16.84	2.52	Eucalyptol	1031	MO
10	18.19	2.44	gamma‐Terpinene	1060	MH
11	20.4	0.91	Linalool	1097	MO
12	24.26	0.14	Borneol	1169	MO
13	24.6	0.8	Terpinen‐1‐ol	1177	MO
14	25.4	0.72	alpha‐Terpineol	1188	MO
15	26.86	0.45	Thymol, methyl ether	1235	MO
16	27.28	0.98	Carvacrol, methyl ether	1244	MO
17	29.46	0.23	Bornyl acetate	1285	MO
18	30.04	26.48	Thymol	1290	MO
19	30.45	38.71	Carvacrol	1299	MO
20	32.23	1.01	Thymol acetate	1352	MO
21	33.1	1.69	Carvacrol acetate	1372	MO
22	35.34	1.35	Caryophyllene <(E)‐>	1419	SH
23	36.16	0.93	Aromadendrene	1441	SH
24	38.35	0.45	Viridiflorene	1496	SH
25	42	0.62	Spathulenol	1578	SO
26	42.2	1.23	Caryophyllene oxide	1583	SO
		99.66	Total Identified		

Abbreviations: MH, monoterpene hydrocarbons; MO, oxygenated monoterpene; SH, sesquiterpene hydrocarbons; SO, oxygenated sesquiterpenes.

### 3.2 Characterization of Nanoparticles

**FIGURE 1:**
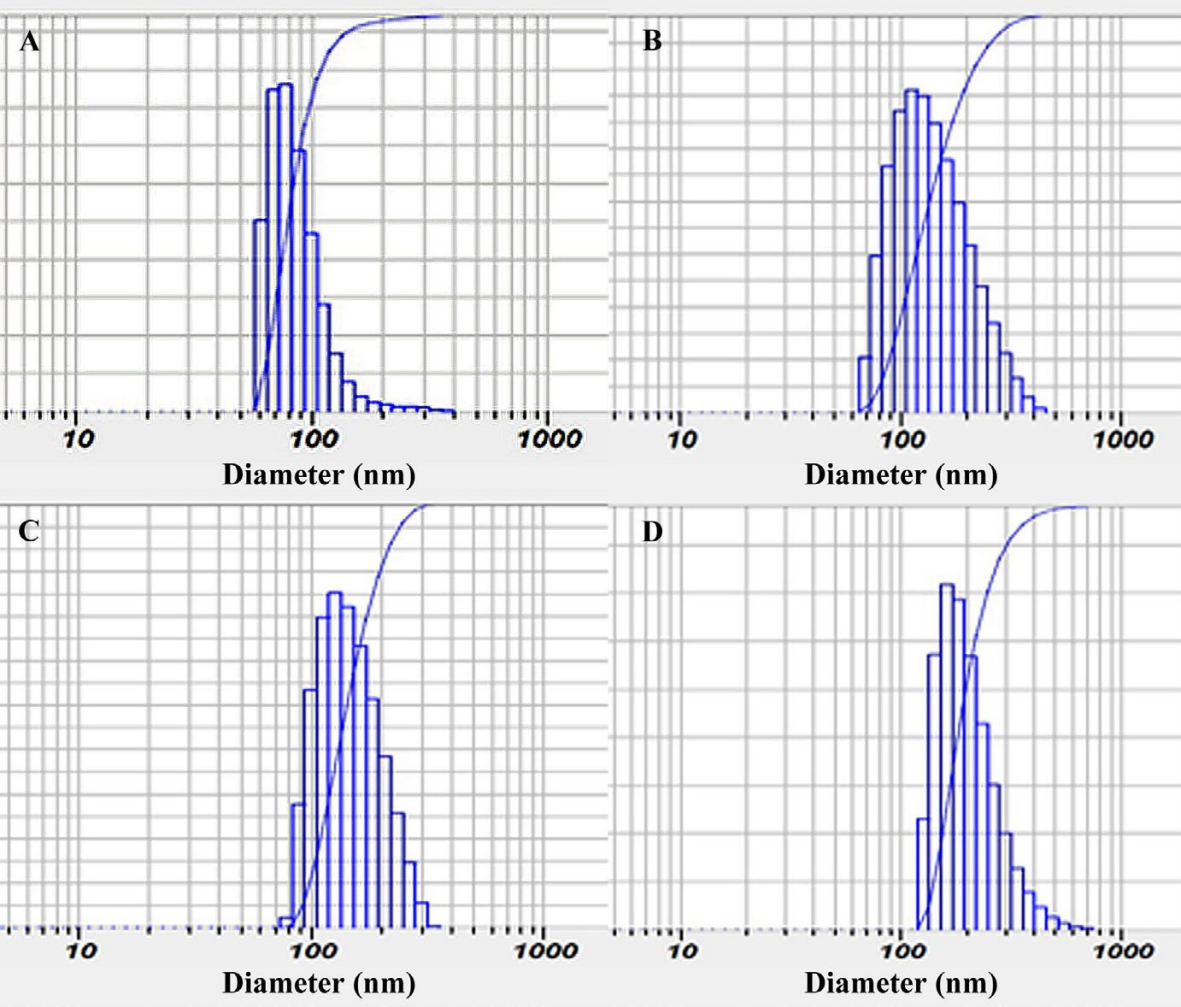
DLS analysis of samples; (A) AlgNPs, (B) AlgNPs–EO, (C) AlgNPs–menthol, and (D) AlgNPs–menthol–EO.

**FIGURE 2:**
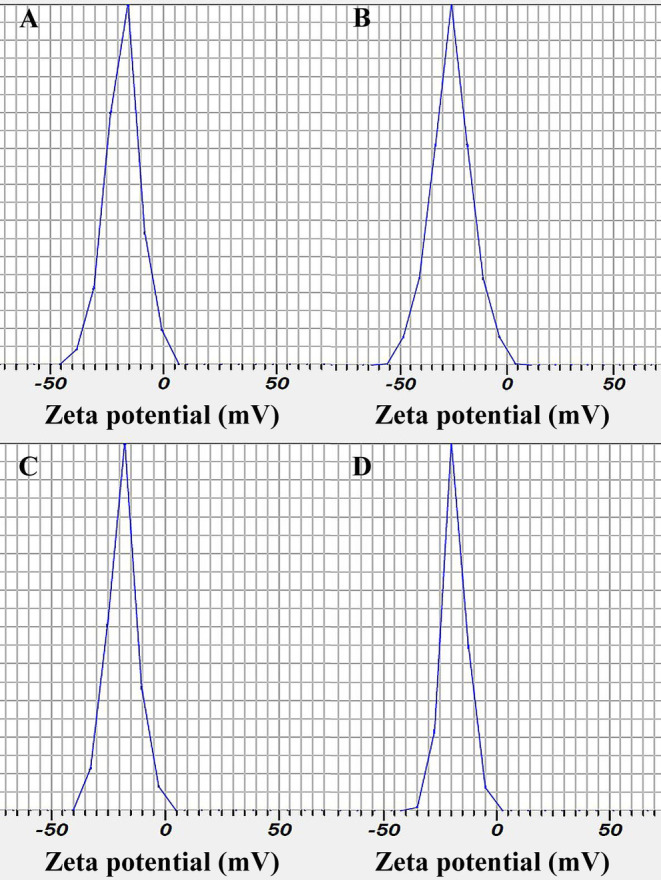
Zeta potentials of (A) AlgNPs, (B) AlgNPs–EO, (C) AlgNPs–menthol, and (D) AlgNPs–menthol–EO.

DLS analysis of nanoparticles is depicted in Figure 1A–D. The particle size of AlgNPs, AlgNPs–EO, AlgNPs–menthol, and AlgNPs–menthol–EO were obtained as 87 ± 4, 146 ± 6, 149 ± 8, and 205 ± 5 nm, respectively. Their PDI values were also 0.17, 0.17, 0.26, and 0.16, and their SPAN values were obtained as 0.44, 0.83, 0.83, and 0.99, respectively. Moreover, their zeta profile of nanoparticles is shown in Figure 2A–D. The values were obtained as −18.8 ± 0.8, −25.8 ± 0.5, −19.6 ± 1.1, and −17.4 ± 0.5 mV, respectively. In the previous studies, Abbasi et al. (2021) reported the particle size and PDI of 176.6 and 184.7 nm and 0.248 and 0.255 for their nanoemulsion of *Z. multiflora* EO and nanoemulsion of *Z. multiflora* EO fortified with cinnamaldehyde, respectively. Another investigation by Alipanah et al. (2022) documented a chitosan nanoparticles conataining *Z. multiflora* with a mean particle size of 177 nm and a SPAN of 0.96. Moreover, Bahrami et al. (2023) reported a range of zeta potential from 16.85 to 21.59 with increasing *Z. multiflora* concentration in gliadin electrospinning loaded with this EO. It was seen in our previous study that, alginate nanoparticles containing *Z. multiflora* and *C. cyminum* EO show size, PDI and zeta potential of 195 nm, 0.109, and −29 mV, respectively. The differences in these studies can be caused by dissimilarities in the ultrasound intensity, preparation time, concentration and type of EO, etc. (Osanloo et al. 2023).

### 3.3 Confirming Successful Loading of the EO and Menthol in AlgNPs by ATR‐FTIR Analysis

**FIGURE 3:**
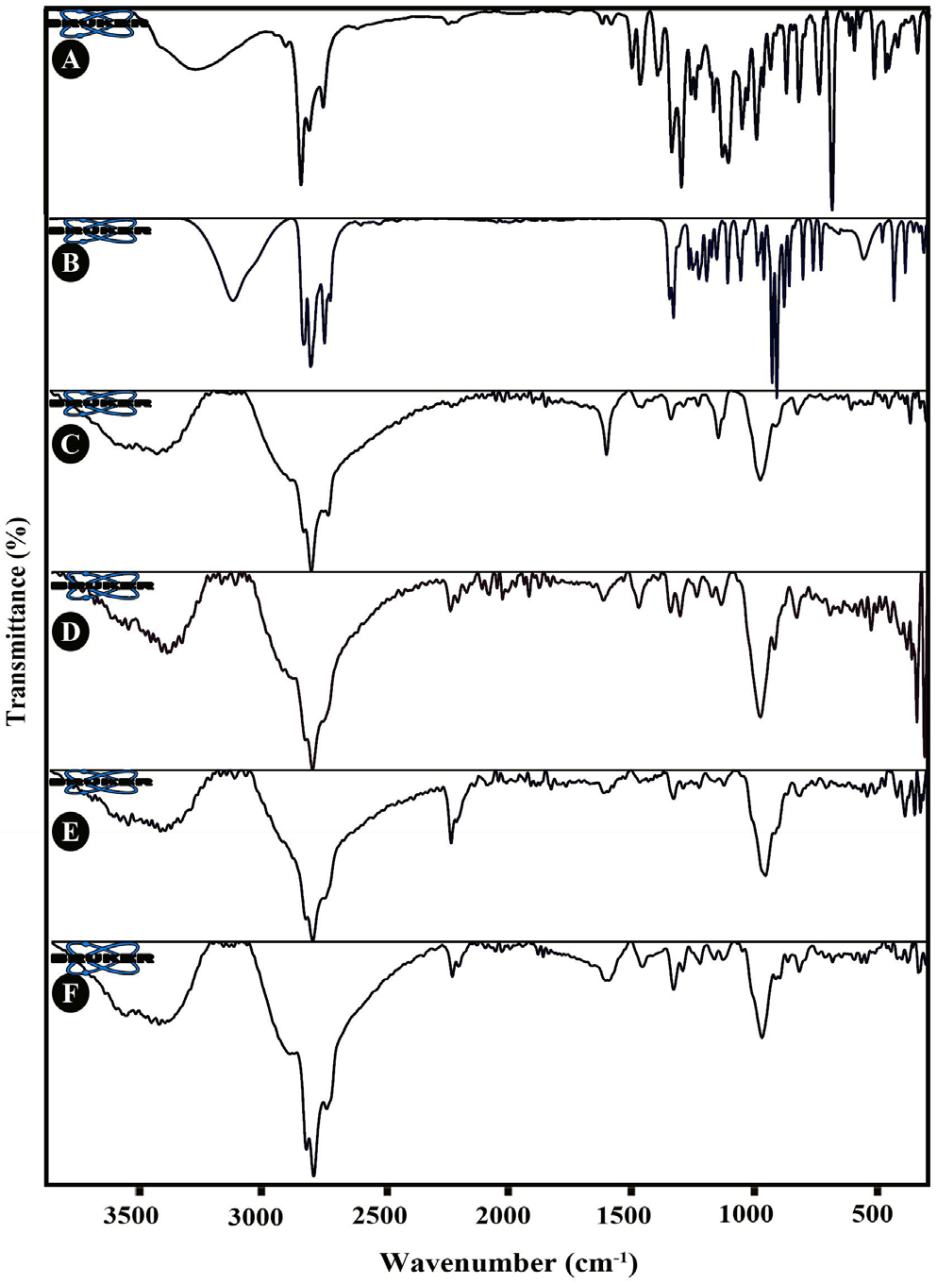
ATR‐FTIR spectra of (A) *Z. multiflora* EO, (B) menthol, (C) AlgNPs, (D) AlgNPs–EO, (E) AlgNPs–menthol, and (F) AlgNPs–menthol–EO.

ATR‐FTIR spectra of the *Z. multiflora* EO, menthol, alginate, AlgNPs, AlgNPs–EO, AlgNPs–menthol, and AlgNPs–menthol–EO are exhibited in Figure 3. The previous study demonstrated that the ATR‐FTIR spectrum of sodium alginate indicated characteristic absorption bands concerning ether, hydroxyl (OH), and carboxylic acid (COOH) functional groups (Li et al. 2016). A broad peak attributed to stretching vibrations of hydroxyl (‐OH) groups was observed at around 3246 cm^−1^. The absorption bands located at 2838–2941 cm^−1^ are assigned to stretching vibrations of aliphatic C–H. Feature peaks at 1404 and 1596 cm^−1^ can be assigned to symmetric and asymmetric stretching vibrations of carboxylate anions (‐COO‐), respectively. The observed peaks at 1716–1792 cm^−1^ indicate stretching vibrations of C=O. The absorption peaks at 1318 and 1029 cm^−1^ can be associated with C–C–H deformation and C–C and C–O stretching vibrations of pyranose rings, respectively. Moreover, the peaks located at around 818 and 941 cm^−1^ can be associated with mannuronic and guluronic acids in the structure of sodium alginate, respectively (Diep and Schiffman 2021; Kuczajowska‐Zadrożna, Filipkowska, and Jóźwiak 2020). *Z. multiflora* EO‐related ATR‐FTIR spectra showed an intense band at 809.58 cm^−1^ due to the overlapping of p‐cymene and thymol bands and indicated the complex composition of *Z. multiflora* EO. This band can be assigned to out‐of‐plane (‐CH) wagging vibrations. The broad absorption peak located at 3385 cm^−1^ is indicative of the stretching vibration of hydroxyl (‐OH) groups. The strong peaks at 2959, 2925, and 2869 cm^−1^ correspond to C–H stretching vibrations in aliphatic groups. Additionally, several highlighted picks can be observed in the wavenumber ranges of 1229–944 cm^−1^ and 862–809 cm^−1^, assigned to in‐plane and out‐of‐plane bending of aromatic C–H, respectively. Other characteristic peaks observed at 1343–1253 cm^−1^ (bending of the O–H groups), 1152 and 1058 cm^−1^ (stretching of the C–OH), 738–592 cm^−1^ (O–H out‐of‐plane bending) (Ahmadi and Jafarizadeh‐Malmiri 2021; Elghobashy et al. 2022; Hu et al. 2018). In the ATR‐FTIR spectrum of menthol, several highlighted peaks are present: the characteristic absorption peak at 3242 cm^−1^ due to O–H stretching vibration, C–H stretching at 2845–2954 cm^−1^, C–O stretching at around 1025–1044 cm^−1^. The 974, 918, 876, 845, and 670 cm^−1^ peaks indicate ring skeleton vibration (Holz et al. 2018; Mossotti et al. 2015; Yingngam et al. 2019). Based on the results obtained from the ATR‐FTIR spectra of alginate and AlgNPs, significant differences can be seen in the width and frequency of their peaks. ATR‐FTIR spectrum of AlgNPs exhibited a broad peak at around 3553 cm^−1^ assigned to the stretching vibration of hydrogen‐bonded O–H (Sarmento et al. 2006). It is noteworthy that the asymmetrical and asymmetrical stretching vibrations of the carboxylate ion (1596 and 1404 cm^−1^) are shifted to higher wavenumbers at 1724 and 1412 cm^−1^, respectively, confirming that calcium ion (Ca^2+^) crosslinked alginate at ‐COO‐ groups (Daemi and Barikani 2012; Li et al. 2016). The peaks at 2925, 2856, and 1462 cm^−1^ are attributed to the asymmetric stretching of C‐H, symmetric stretching of C‐H, and bending vibrations of C‐H, respectively. The peaks that appeared at 1351, 1098, and 1036 cm^−1^ are assigned to a secondary alcoholic group, stretching vibration of C‐O‐C in the pyranosyl ring and stretching vibration of C‐O. The 488 and 576 cm^−1^ absorption peaks indicate C‐C skeletal vibrations and C‐H out‐of‐plane bending (Larosa et al. 2018). In the ATR‐FTIR spectrum of *Z. multiflora* EO‐loaded alginate nanoparticles (AlgNPs–EO), the fingerprint region (638–1353 cm^−1^) seems richer in characteristic peaks than that of blank nanoparticles. Moreover, changes in the position and intensity of some absorption peaks indicate significant interactions among alginate, *Z. multiflora* EO, and Ca^2+^. The hydroxyl groups of *Z. multiflora* EO could probably make intermolecular hydrogen bonds with alginate and share it with the calcium ion (Ca2+) coordination site. ATR‐FTIR spectrum of AlgNPs–EO also exhibited a broad peak at around 3486 cm^−1^ corresponding to hydroxyl (–OH) stretching vibration and hydrogen bonding. The peak at 2926 cm^−1^ is assigned to the stretching vibration of C–H in an aromatic compound. The presence of *Z. multiflora* EO in alginate nanoparticles is also confirmed by the absorption peaks at 1590, 1353, and 1252 cm^−1^. In the ATR‐FTIR spectrum of the menthol‐loaded alginate nanoparticles (AlgNPs–menthol), the characteristic peaks of menthol located at around 1025–1044 cm^−1^ disappeared, confirming that menthol was loaded into the alginate nanoparticles (Yingngam et al. 2019). The characteristic band at 3242 cm^−1^ corresponding to the hydroxyl group (‐OH) in menthol shifted to 3543 cm^−1^ in the ATR‐FTIR spectrum of AlgNPs–menthol because of possible hydrogen bonding. In the case of *Z. multiflora*/menthol loaded alginate nanoparticles (AlgNPs–menthol–EO), our ATR‐FTIR results showed that characteristic peaks of the main components were present, thus indicating the successful loading of *Z. multiflora* EO and menthol in the final nanoparticles.

### 3.4 Chemical Analysis of Chicken Thigh Samples During Cold Storage

#### 3.4.1 Determination of pH Changes

**FIGURE 4:**
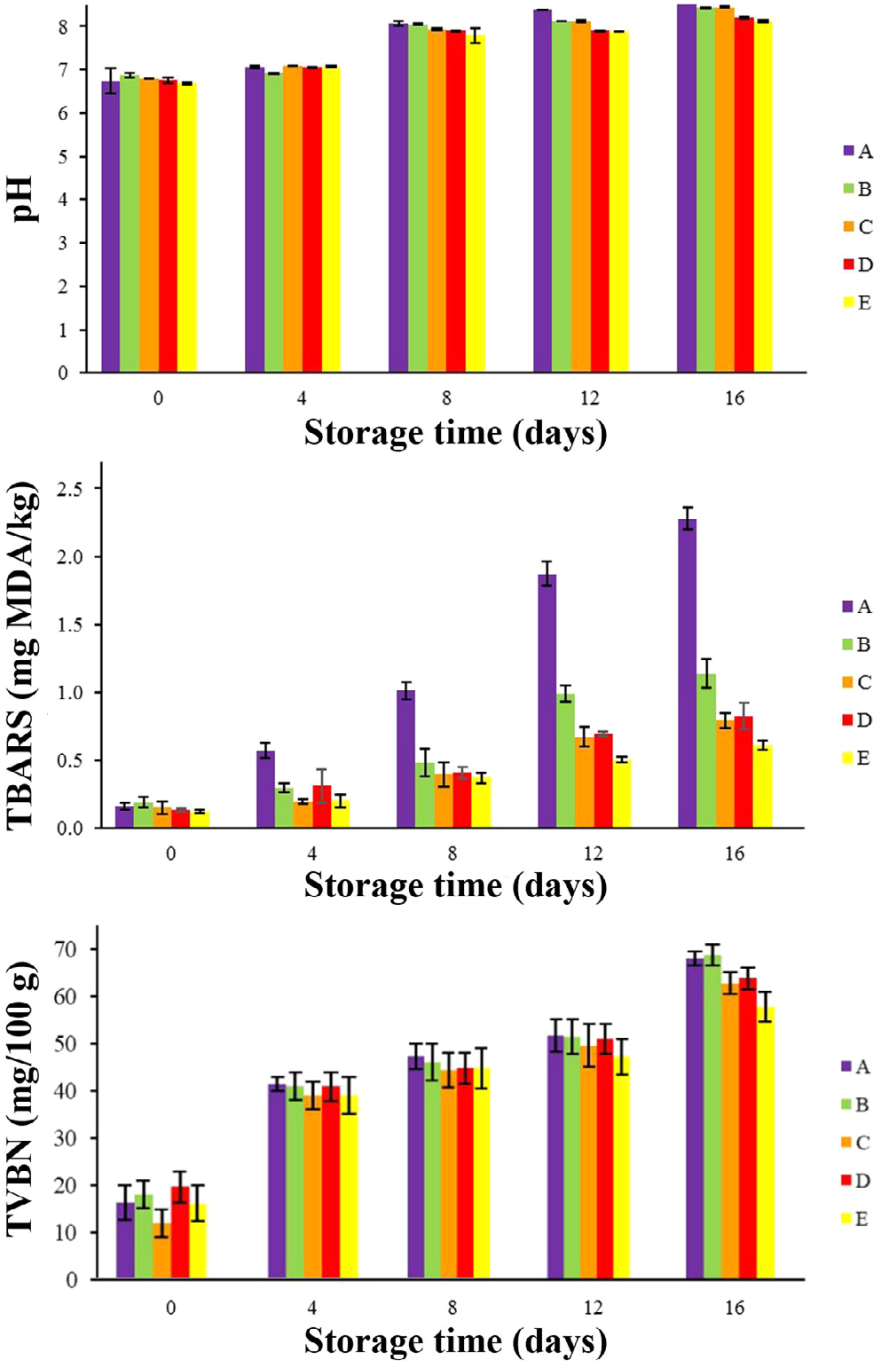
PH, TBARS, and TVBN changes of chicken thigh samples during refrigerated storage. *p* < 0.05. (A: Control. B: AlgNPs, C: AlgNPs–menthol, D: AlgNPs–EO, and E: AlgNPs–menthol–EO).

Figure 4 (the first part) depicts the changes in pH values for chicken thigh samples during cold storage. The initial pH values of the samples ranged from 6.68 to 6.86 (day 0). The pH values of chicken thigh significantly (*p* < 0.05) increased during refrigerated storage, with the control group exhibiting the highest increment (reached to 8.64 on day 16). In contact coated samples containing nanoparticles of both menthol and *Z. multiflora* EO (AlgNPs–menthol–EO) showed the lowest pH increase rate (8.12 on day 16), among other samples, probably due to effective suppressed bacterial growth and minimized endogenous alkalinizing reactions. The autolytic reactions of native enzymes and the proteolytic activities of various bacteria, which result in the creation of basic nitrogenous components such ammonia and biogenic amines, are likely the cause of the pH growing trend. These reactions might suggest the circumstances leading to chemical degradation. But the equilibrium of alkaline materials and the lactic acid produced by bacteria via storage time allowed the chicken samples to reach their final pH. The results indicate that samples with *Z. multiflora* EO, menthol, or both exhibited a lower pH increase than samples without these components. This is likely due to the antibacterial and antioxidant properties of these compounds (Baek, Lee, and Oh 2021; Majdinasab et al. 2020). It can be also seen from the findings that *Z. multiflora* EO was more effective than menthol in this regard.

These findings corroborate the results of previous studies (Baek, Lee, and Oh 2021; Mojaddar Langroodi, Nematollahi, and Sayadi 2021; Osanloo et al. 2023) which studied the effect of alginate nanoparticles coating containing *Z. multiflora* and *Cuminum cyminum* EOs, chitosan coating containing grape seed extract and *Origanum vulgare* EO, and alginate‐coating containing grapefruit seed extract on pH of shrimp, turkey meat, and shrimp, respectively. Other related studies also found that the active edible coating substantially inhibited the pH rising during food storage (Abbasi et al. 2021; Bahmani and Abolfathi 2022; Hashemi, Dastjerdi, et al. 2021).

#### 3.4.2 Assessment of TBARS Changes

The oxidation of unsaturated fatty acids in meat leads to the formation of secondary products, including hydroperoxides and peroxides, which ultimately produce malondialdehyde (MDA), a marker of lipid oxidation and rancidity (Huang et al. 2020). Lipid oxidation adversely affects meat's sensory, functional, and nutritional properties, consequently influencing its shelf life. It is important to recognize that lipid peroxidation contributes to developing undesirable off‐flavors in meat products containing PUFA (polyunsaturated fatty acid) during storage. The TBARS value is a widely used index for assessing the extent of lipid oxidation and the MDA content in meat (Mojaddar Langroodi, Nematollahi, and Sayadi 2021; Osanloo et al. 2023). The results of TBARS values of all samples are shown in Figure 4 (the second part). According to the figure, the TBARS amount of all samples, similar to pH, has increased from day 0 to 16. On day 0, all the results are close to each other (0.12–0.19 mg MDA/kg), but the values become different over time. On day 16, the highest value is related to the control sample (2.28 mg MDA/kg), and the lowest value is related to the AlgNPs–menthol–EO sample (0.61 mg MDA/kg). In general, from 4 to 16 days, after the AlgNPs–menthol–EO sample, the AlgNPs–menthol sample is in second place, and AlgNPs–EO is in third place, which indicates the greater antioxidant power of menthol compared to *Z. multiflora* EO. However, there was no significant difference between menthol and *Z. multiflora* EO (*p* > 0.05), at the end of cold storage. The significantly lower TBARS values in the AlgNPs–menthol–EO samples, in all time intervals, can be attributed to the presence of potent antioxidant compounds derived from *Z. multiflora* EO (thymol and carvacrol) and menthol. These compounds are known to effectively scavenge free radicals and active chelating elements like iron, thereby contributing to the preservation of chicken thigh quality (Alipanah et al. 2022). A TBARS value of 2–5 mg MDA/kg is generally considered the threshold for humans to perceive off‐flavor in meat (Mojaddar Langroodi, Nematollahi, and Sayadi 2021). The TBARS value of all samples is below 2, except for the control sample on the 16th day. This indicates the potential of these compounds to counteract oxidative degradation in meat. These findings corroborate the results of previous studies (Huang et al. 2020; Mehdizadeh and Langroodi 2019; Raeisi et al. 2020).

#### 3.4.3 Assessment of TVBN Changes

Total volatile basic nitrogen (TVBN), a measure of protein degradation and microbial activity, is a widely used indicator of meat and meat product quality. During storage, endogenous enzymes and microorganisms contribute to increased TVBN levels due to protein breakdown, ammonia production, and other volatile nitrogen compounds. Through enzymatic mechanisms, microorganisms catalyze the conversion of trimethylamine oxide (TMAO) to trimethylamine (TMA), a volatile nitrogenous molecule (Mojaddar Langroodi, Nematollahi, and Sayadi 2021). Therefore, applying antioxidants and antimicrobial agents can effectively inhibit lipid oxidation and microbial growth, minimizing TVBN accumulation during storage (Raeisi et al. 2020). The results of TVBN values of all samples are shown in Figure 4 (the third part). According to the figure, the amount of this value in all samples, similar to pH and TBARS, has increased from day 0 to 16. As it is clear in this figure, the increasing trend of this index was the highest in the control sample, compared to coated treatments; so that at the end of the storage time it reached to 67.98 mg N/100 g. Alginate nanoparticles with menthol‐EO, on the other hand, had the lowest TVBN level on day 16 (57.81 mg N/100 g). These findings indicate that the combination of menthol and *Z. multiflora* EO substantially impacts TVBN content compared to samples containing *Z. multiflora* EO or menthol alone, probably due to these compounds' combined antibacterial and antioxidant properties. This large accumulation of nitrogenous bases was probably caused by the higher microbial populations seen in the control treatment. Furthermore, a positive association was found between TVBN and pH values, indicating that elevated volatile amine levels corresponded with higher pH values (Sayyari et al. 2021). These findings are in line with previous studies (Baek, Lee, and Oh 2021; Hashemi, Dastjerdi, et al. 2021; Hashemi, Shakerardekani, et al. 2021; Huang et al. 2020; Liu et al. 2020). The study by Majdinasab et al. (2020), in contrast to the findings of the present investigation, did not find a synergistic effect between thyme and summer savory EOs on preventing the chemical changes in coated chicken fillets with basil seed gum. This is likely due to the variations in EOs and their concentrations as well as their sizes. According to the results, it can be also understood that the effect of menthol on TVBN is greater than *Z. multiflora* EO, in all time intervals.

### 3.5 Microbiological Analysis

**FIGURE 5:**
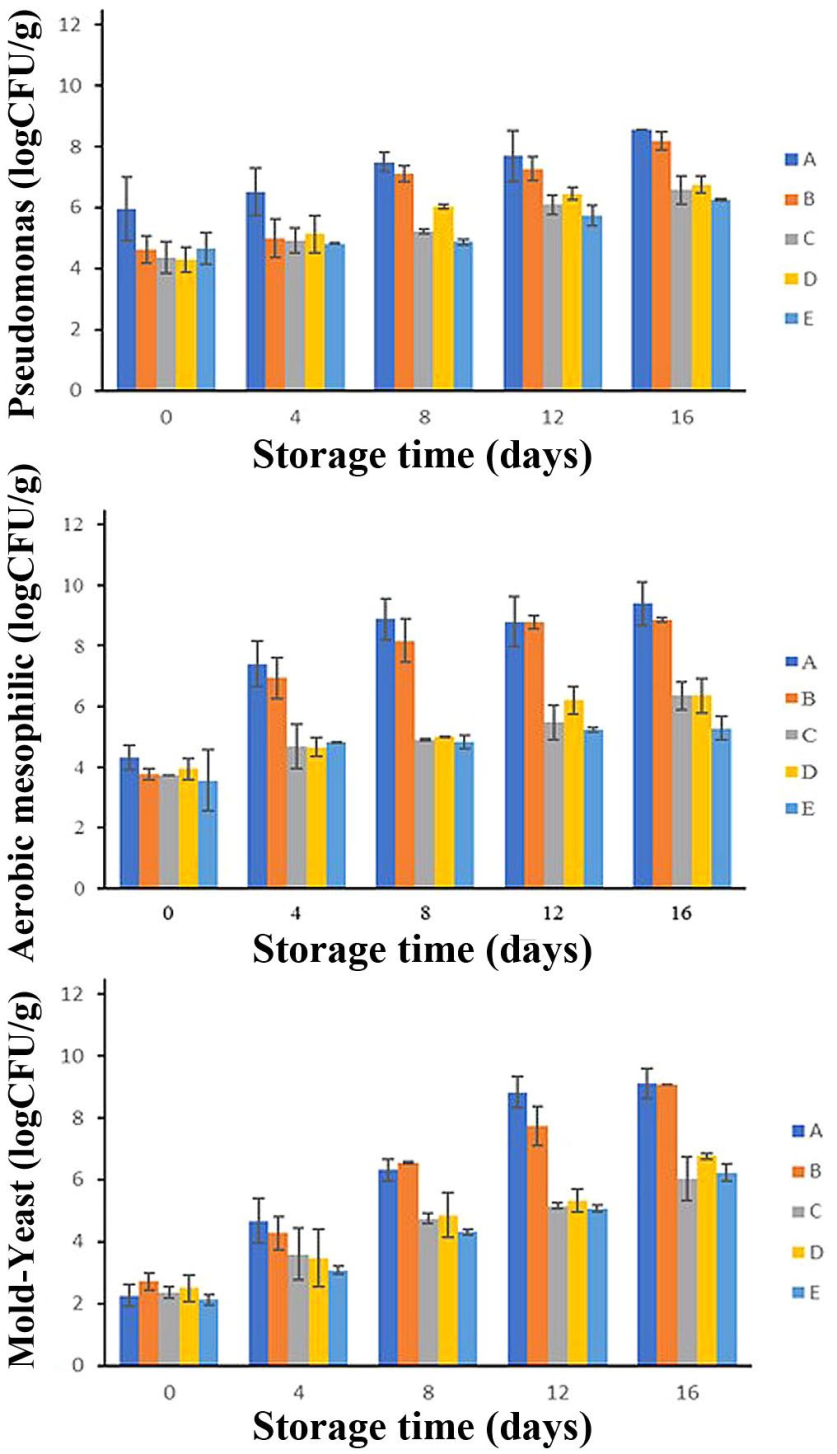
Microbial analysis changes of chicken thigh samples during refrigerated storage. *p* < 0.05. (A: Control. B: AlgNPs, C: AlgNPs–menthol, D: AlgNPs–EO, and E: AlgNPs–menthol–EO).

Figure 5 depicts the changes in Pseudomonas, aerobic mesophilic, and yeast‐mold counts of chicken thigh meat samples over 16 days of refrigerated storage at 4°C. As can be seen in this figure, the trend of changes in the number of microorganisms during cold storage was also increasing, just like the chemical indicators (pH, TBARS, and TVBN). The highest increasing trend in all treatments was related to the number of mold and yeast and the lowest was related to the Pseudomonas count. Spoilage of fresh meat is primarily caused by the activity of specific spoilage microorganisms, generating metabolites that lead to off‐flavors and off‐odors, ultimately resulting in consumer rejection. The shelf life of various types of meat, including fresh poultry meat, is typically limited by microbial spoilage, which is prevalent in spoiled meat environments (Mojaddar Langroodi, Nematollahi, and Sayadi 2021).

#### 3.5.1 Pseudomonas Count

Pseudomonas species are a significant component of the meat microflora, characterized as strictly aerobic bacteria that require an abundant oxygen supply for survival. The spoilage of meat products, stored under aerobic refrigerated conditions, is primarily attributed to the growth and metabolic activities of Pseudomonas species, which can degrade amino acids and glucose even at low temperatures. The proteolytic activity of these bacteria is likely responsible for meat spoilage and the subsequent production of slime. It has been reported that if the number of Pseudomonas in meat exceeds 7, spoilage is evident in this product (Osanloo et al. 2023). Figure 5 (the first part) shows the changes in the number of Pseudomonasin chicken thigh samples during refrigeration. The number of Pseudomonas in the control sample showed a stronger increase compared to the coated treatments (from 5.97 to 8.57 Log CFU/g, during 16 days of refrigerated storage). The best result is related to the AlgNPs–menthol–EO sample, which has grown from 4.29 Log CFU/g (day 0) to 6.26 Log CFU/g (day 16). From the comparison of AlgNPs–menthol and AlgNPs–EO sample results, it can be concluded that menthol had a better effect on limiting the growth of Pseudomonas.

The results obtained in this study are confirmed by other studies, including Bahmani and Abolfathi (2022), who investigated the shelf life of chitosan‐coated chicken fillets containing *Z. multiflora* EO. Furthermore, a study done by Mehdizadeh and Langroodi (2019) revealed that the number of Pseudomonas bacteria in chicken breast meat samples increased significantly during cold storage. However, samples coated with chitosan incorporating propolis extract and *Z. multiflora* EO exhibited substantially lower bacterial counts, cleaerlyattributable to the antimicrobial effect of these EOs during the storage period.

The antimicrobial potential of *Z. multiflora* EO and menthol is largely attributed to their phenolic compounds, which sensitize the phospholipids in the bacterial cell membrane, increasing permeability and causing the leakage of essential intracellular components or damaging their enzyme structures. They can effectively break down the outer membrane of Gram‐negative bacteria (like Pseudomonas), possibly through a mechanism like cation chelation, leading to the breakdown of the ATP energy supply. However, EOs are more effective against Gram‐positive bacteria by causing phosphate ion leakage in their membrane. Also, the synergistic effects of EOs and different extracts, like menthol, are not fully understood. However, menthol likely inhibit bacteria by increasing the number and size of pores in cell membranes (Mojaddar Langroodi, Nematollahi, and Sayadi 2021; Osanloo et al. 2023). Therefore, the mechanism of alginate nanoparticles containing *Z. multiflora* EO and menthol against Pseudomonas is primarily related to the fact that thymol, carvacrol, and menthol most likely cause the breakdown of bacterial membranes, leading to cytoplasmic leakage, cell lysis, and ultimately cell damage (Hashemi, Shakerardekani, et al. 2021). Our findings also revealed that the AlgNPs sample did not effectively reduce bacterial growth. However, adding *Z. multiflora* EO and menthol (AlgNPs–EO–menthol samples) increased their ability to limit bacterial contamination. It has been shown that the nanoparticles of EO has a higher antimicrobial effect than traditional, possibly due to the reduced EO particle size and increased diffusion of active agents into microorganisms (Osanloo et al. 2023).

#### 3.5.2 Aerobic Mesophilic Count

Fresh chicken meats are extremely susceptible to bacterial infection from certain points in the production process until they are consumed. For this reason, most safety regulations provide for regulating the aerobic mesophilic bacteria count (Majdinasab et al. 2020). Figure 5 (the second part) shows the aerobic mesophilic count in chicken thigh samples during refrigeration. As depicted in this figure, the control sample had the worst result on all days, increasing from 4.31 (day 0) to 9.4 Log CFU/g (day 16). The most antimicrobial effect is related to the AlgNPs–menthol–EO sample, which has grown from 3.56 (day 0) to 5.28 Log CFU/g (day 16). With comparison of AlgNPs‐menthol and AlgNPs–EO samples results, it can be concluded that *Z. multiflora* EO is more effective than menthol in preventing the growth of aerobic mesophiles. This is presumably because different extracts have varied effects on different microbial groups. Iranian Veterinary Organization has set the permissible limit of mesophilic bacteria in chicken meat to 7 Log CFU/g (Bahmani and Abolfathi 2022). It can be seen in the figure that only the control and AlgNPs samples exceed the permissible limit from the fourth day, and the rest of the samples are within the permissible limit until the last day of storage. Numerous research have found similar findings regarding the antibacterial potential and shelf‐life extending capacity of EOs on the nanoscale in various foods (Abbasi et al. 2021; Elghobashy et al. 2022; Huang et al. 2020; Osanloo et al. 2023; Rahnemoon et al. 2021; Sayyari et al. 2021). Moreover, Hashemi et al. confirmed the aerobic mesophilic results of this study, which reported that samples treated with 1% alginate enriched with *Z. multiflora* EO (0.3%) showed significantly less number than the control (Hashemi, Dastjerdi, et al. 2021). Also, Osanloo et al. reported that The AlgNPs coating containing thyme and cumin EO exhibited consistently lower counts of aerobic mesophilic bacteria across all storage durations, demonstrating their effectiveness in inhibiting bacterial growth during refrigerated storage of shrimp samples (Osanloo et al. 2023).

Thus, it is possible to argue that the combination of *Z. multiflora* EO and menthol may increase the cytoplasmic membrane's penetrability due to the potent components of thyme, particularly thymol and carvacrol which facilitate the easier uptake of menthol by the cell. Furthermore, by mixing menthol with proteins in the cell membrane, thymol and carvacrol may expand the size of the pores' presence and provide synergistic effects. It has been well‐documented that EOs are effective antibacterial agents against a wide range of pathogens. The main source of this powerful action is thought to be the phenolic chemicals found in the EOs. Broad‐spectrum antibacterial action against a variety of pathogens (both Gram‐positive and Gram‐negative bacteria) is demonstrated by these phenolic compounds. Their capacity to compromise the integrity of bacteria's cell membranes is probably what gives them their effectiveness (Abbasi et al. 2021). Potential antimicrobial methods may involve the chelation of critical cations, which can result in ATP depletion, a decrease in intracellular pH, and a decrease in potassium levels. These processes may have an additional negative impact on the viability of the bacteria by making the cell membrane more permeable and fluid, which could allow essential lipid and protein components to leak out and ultimately cause cell death [32]. Due to their large surface area and trivial droplet size, *Z. multiflora* EO and menthol nanoparticles have a significant impact on the cell membrane and, as a result, exhibit good molecular interactions with multiple locations inside the microbial cell membrane (Abbasi et al. 2021; Osanloo et al. 2023).

#### 3.5.3 Yeast and Mold Count

Several studies have shown that yeast‐mold species are also involved in chicken meat spoilage (Huang et al. 2020; Rahnemoon et al. 2021). Figure 5 shows the growth graph of yeast and mold in chicken thigh samples during refrigerated storage. The initial amount of mold and yeast on the first day varied between 2.13 and 2.72 Log CFU/g. With the passage of time in the refrigerator, like the bacteria groups, an increasing trend was observed in the amount of yeast and mold. The maximum amount, on day 16, belongs to the control sample (9.08 Log CFU/g) and the lowest number correspond to coated samples containing both *Z. multiflora* EO and menthol (AlgNPs–menthol–EO) which reached to 6.24 Log CFU/g, at the end of storage. Moreover, the results depicted that menthol has a greater effect on inhibiting the growth of mold and yeast compared to thyme, similar to Pseudomonas graph. *Z. multiflora* EO could suppress the colony's size and sporulation. In addition, the morphological changes of fungi, destruction of the hyphae, vacuolization of cytoplasm, cell swelling, detachment of the cell membrane from the cell wall, deformation of mycelia, and shedding the cytoplasm from the cell were the main alterations caused by thyme EO. Thus, it could be said that the main sites of action of EO are the plasma membrane and cell wall. In conclusion, morphological and structural changes may be one of the mechanisms involved in the growth inhibition of fungi (Shokri and Sharifzadeh 2016). It is worthy to mention that menthol and thyme EO or their combination could inhibit the growth and spore developmemt of food‐infesting fungi and could be recommended as plant‐based food preservatives to improve the shelf life of stored food products (Ben Miri et al. 2023). Some studies have shown the effect of thyme or menthol on preventing the growth of mold and yeast (Abbaszadeh, Sharifzadeh, and Mahmoodzadeh Hosseini 2019; Ben Miri et al. 2023; Gandomi et al. 2009; Mohammadi, Hashemi, and Hosseini 2015; Nakhaee Moghadam et al. 2021; Yahyaraeyat et al. 2013). Similar to this study, Mojaddar Langroodi, Nematollahi, and Sayadi (2021) reported that the highest counts of yeast and mold were found in the control treatment, and the lowest numbers were observed in combined treatment, containing both grape seeds extract and oregano EO, which were in good agreement with Rahnemoon et al. (2021), Huang et al. (2020) studies.

### 3.6 Sensory Evaluation

**FIGURE 6:**
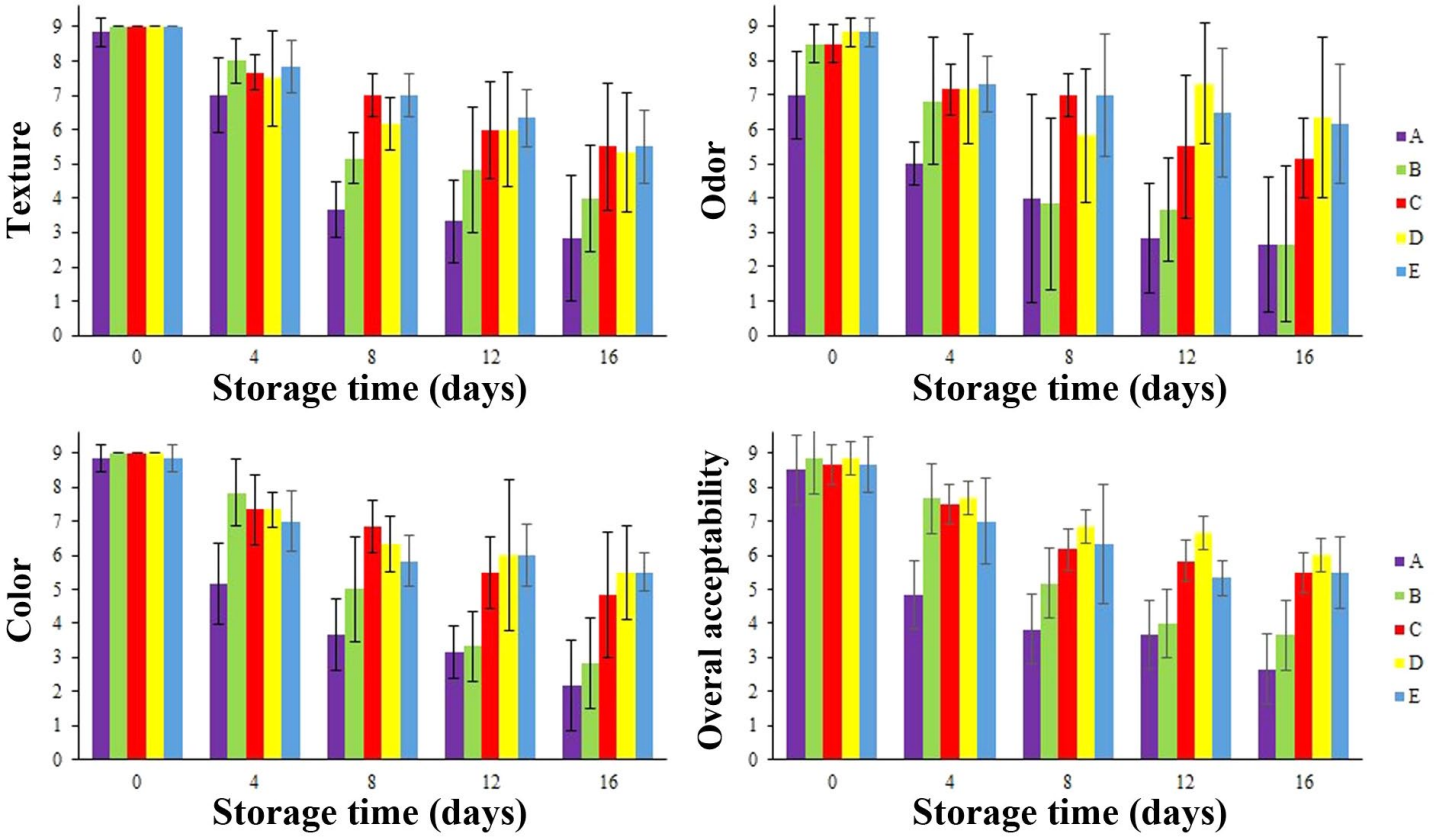
Sensory evaluation on texture, odor, color, and overall acceptability during refrigerated storage. *p* < 0.05. (A: Control. B: AlgNPs, C: AlgNPs–menthol, D: AlgNPs–EO, and E: AlgNPs–menthol–EO).

Figure 6 depicts the changes in sensory attributes (texture, odor, color, and overall acceptability) of different treatments of chicken thigh meat throughout the storage period at 4°C. All samples were evaluated using a nine‐point hedonic scale, with scores below 7 indicating unacceptable sensory quality for consumers (Mehdizadeh and Langroodi 2019). As shown in Figure 6, the sensory scores exhibited a significant decline (*p* < 0.05) in all samples during the 16‐day cold storage period, attributed to microbial activities and chemical alterations induced by the treatments. Across all sensory attributes, the sharpest score decline was observed for the control and AlgNPs treatments, dropping from approximately 9 (day 0) to around 2–4 (day 16). These treatments also exhibited the lowest acceptability scores up to day 4 of storage.

In contrast, the other three coated treatments displayed a milder score decline, ranging from approximately 9 to about 5–6. These treatments maintained acceptable sensory scores up to day 4 or 8, with the AlgNPs–Menthol and AlgNPs–Menthol–EO treatments even maintaining acceptable texture and odor scores up to day 8. Notably, a sensory score for odor was recorded for the AlgNPs–EO treatment on day 12, even though no acceptable scores were obtained for day 8. This may be attributed to the testers' fluctuating mental and physical states. It is worth emphasizing that the outcomes of the sensory assessment are partially linked to chemical and microbial evaluations. Hence, it can be inferred that including menthol and *Z. multiflora* EO, owing to their antioxidant and antimicrobial properties, could exert a protective effect against chemical and microbial changes, thereby mitigating their detrimental impact on sensory properties. Our results agree with (Hematizad et al. 2021) and (Baek, Lee, and Oh 2021) studies. Also, with the increase in pH, all samples maintained values below the acceptable limit for sensory acceptability, further emphasizing the limitations of pH as a single indicator of food quality. These results also show that, among these 4 sensory values, the highest score decrease was observed in the color parameter (a mean reduction of 5 points), followed by texture (a mean reduction of 4.33 points). Also, in terms of organoleptic indicators, except texture, the score of *Z. multiflora* EO was higher than that of menthol‐containing samples.

## 4 Conclusions

The present study demonstrated the efficacy of alginate nanoparticles incorporating *Z. multiflora* EO and menthol in combating the proliferation of various spoilage microorganisms, including Pseudomonas spp., aerobic mesophilic bacteria, and yeast mold counts. It also delayed lipid oxidation, preserved sensory quality, and extended the shelf life of fresh chicken thigh meat during refrigerated storage. Therefore, alginate nanoparticles containing *Z. multiflora* EO and menthol emerge as promising coating agents for preserving chicken meat under refrigerated conditions.

Our study clearly shows the amazing potential of alginate nanoparticles loaded with *Z. multiflora* EO and menthol, or both, as an active packaging method for keeping fresh chicken tighs. Pseudomonas spp., aerobic mesophilic bacteria, and yeast mold counts are only a few of the spoilage bacteria that this novel method effectively combats with its strong antimicrobial activity. These samples also showed a noticeable delay in lipid oxidation and maintained their sensory qualities over the course of refrigeration (4°C). All of these factors work together to greatly increase the shelf life of fresh chicken thighs. Alginate nanoparticles with menthol and thyme EO appear as a viable new active packaging method for guaranteeing the realistic preservation of fresh meat in cold storage in light of these convincing results.

## Author Contributions

**Ali Ranjbar:** data curation (equal), formal analysis (equal), writing – original draft (equal). **Mahmoud Osanloo:** investigation (equal), methodology (equal), writing – review and editing (equal). **Mojdeh Safari:** investigation (equal), methodology (equal), writing – original draft (equal). **Zahra Eskandari:** formal analysis (equal), methodology (equal), writing – original draft (equal). **Ehsan Safari:** formal analysis (equal), methodology (equal), writing – original draft (equal). **Amene Nematollahi:** investigation (equal), methodology (equal), project administration (equal), supervision (equal), writing – review and editing (equal).

## Conflicts of Interest

The authors declare no conflicts of interest.

## Data Availability

The data that support the findings of this study are available on request from the corresponding author.
